# Epidemiology and hypothetical transmission cycles of *Trichinella* infections in the Greater Kruger National Park of South Africa: an example of host-parasite interactions in an environment with minimal human interactions

**DOI:** 10.1051/parasite/2020010

**Published:** 2020-03-12

**Authors:** Louis J. La Grange, Samson Mukaratirwa

**Affiliations:** 1 Department of Agriculture, Rural Development, Land and Environmental Affairs, Chief Directorate Veterinary Services, Veterinary Public Health Private Bag X11309 Mbombela 1200 South Africa; 2 University of KwaZulu-Natal, School of Life Sciences, Westville Campus Durban 4000 South Africa; 3 One Health Center for Zoonoses and Tropical Veterinary Medicine, Ross University School of Veterinary Medicine P.O. Box 334 St Kitts Basseterre West Indies

**Keywords:** *Trichinella*, Kruger National Park, South Africa

## Abstract

Knowledge on the epidemiology, host range and transmission of *Trichinella* spp. infections in different ecological zones in southern Africa including areas of wildlife-human interface is limited. The majority of reports on *Trichinella* infections in sub-Saharan Africa were from wildlife resident in protected areas. Elucidation of the epidemiology of the infections and the prediction of hosts involved in the sylvatic cycles within specific ecological niches is critical. Of recent, there have been reports of *Trichinella* infections in several wildlife species within the Greater Kruger National Park (GKNP) of South Africa, which has prompted the revision and update of published hypothetical transmission cycles including the hypothetical options based previously on the biology and feeding behaviour of wildlife hosts confined to the GKNP. Using data gathered from surveillance studies and reports spanning the period 1964–2019, confirmed transmission cycles and revised hypothesized transmission cycles of three known *Trichinella* species (*T. zimbabwensis*, *Trichinella* T8 and *T. nelsoni*) are presented. These were formulated based on the epidemiological factors, feeding habits of hosts and prevalence data gathered from the GKNP. We presume that the formulated sylvatic cycles may be extrapolated to similar national parks and wildlife protected areas in sub-Saharan Africa where the same host and parasite species are known to occur. The anecdotal nature of some of the presented data confirms the need for more intense epidemiological surveillance in national parks and wildlife protected areas in the rest of sub-Saharan Africa to unravel the epidemiology of *Trichinella* infections in these unique and diverse protected landscapes.

## Abstract (fr)

Les connaissances sur l’épidémiologie, la gamme d’hôtes et la transmission des infections à *Trichinella* spp. dans différentes zones écologiques d’Afrique australe, y compris les zones d’interface faune-homme, sont limitées. La majorité des signalements d’infections à *Trichinella* en Afrique subsaharienne proviennent animaux sauvages résidant dans des zones protégées. L’élucidation de l’épidémiologie des infections et la prévision des hôtes impliqués dans les cycles sylvatiques au sein de niches écologiques spécifiques sont essentielles. Récemment, des infections à *Trichinella* ont été signalées chez plusieurs espèces sauvages dans le Grand Parc National Kruger (GPNK) en Afrique du Sud, ce qui a incité à la révision et à la mise à jour des cycles de transmission hypothétiques publiés, y compris les options hypothétiques basées précédemment sur la biologie et le comportement alimentaire des hôtes de la faune sauvage confinés au GPNK. À partir des données des études de surveillance et de rapports couvrant la période 1964–2019, les cycles de transmission confirmés et les cycles de transmission hypothétiques révisés de trois espèces connues de *Trichinella* (*T. zimbabwensis*, *Trichinella* T8 et *T. nelsoni*) sont présentés. Ceux-ci ont été formulés sur la base des facteurs épidémiologiques, des habitudes alimentaires des hôtes et des données de prévalence recueillies au GPNK. Nous supposons que les cycles sylvatiques formulés peuvent être extrapolés aux parcs nationaux et zones protégées similaires en Afrique subsaharienne, où les mêmes hôtes et espèces de parasites sont connues. La nature anecdotique de certaines des données présentées confirme la nécessité d’une surveillance épidémiologique plus intense dans les parcs nationaux et zones protégées du reste de l’Afrique subsaharienne pour démêler l’épidémiologie des infections à *Trichinella* dans ces paysages protégés uniques et diversifiés.

## Introduction

Nematodes of the genus *Trichinella* are zoonotic and have a cosmopolitan distribution and infect an array of hosts ranging from cold-blooded reptiles to birds and mammals [53, 65, 70, 81]. Ten species are known to exist within the genus; *Trichinella murrelli* Pozio & La Rosa, 2000 [71], *T. papuae* Pozio et al., 1999 [77], *T. nativa* Britov & Boev, 1972 [6], *T. britovi* Pozio et al., 1992 [73], *T. spiralis* Owen, 1835 [59], *T. pseudospiralis* Garkavi, 1972 [17], *T. nelsoni* Britov & Boev, 1972 [6], *T. patagoniensis* Krivokapich et al., 2012 [34], *T. zimbabwensis* Pozio et al., 2002 [69] and *Trichinella* T13, Sharma, 2019; Sharma et al., 2019 [89, 90], as well as three additional genotypes, *Trichinella* T6 Pozio et al., 1992 [73], *Trichinella* T8 Pozio et al., 1992 [73] and *Trichinella* T9 Nagano et al., 1999 [55]. At least four species of *Trichinella* are known to circulate in sub-Saharan Africa, including *T. nelsoni*, *Trichinella* T8, *T. britovi* and *T. zimbabwensis* [53]. Of the four *Trichinella* species known to circulate in this region, all except *T. britovi,* have been reported in the GKNP [53, 54]. Mukaratirwa et al. [53, 54] confirmed lions (*Panthera leo*) and hyaenas (*Crocuta crocuta*) to be the major reservoirs for *Trichinella* infections in the Greater Kruger National Park (GKNP), based on reported prevalence data. However, of late, *Trichinella* spp. infections have been confirmed in at least six mammalian and two reptile species from the GKNP [38, 40, 43, 54, 79] as well as *Trichinella*-like infections in at least six additional mammalian hosts [43, 54, 100, 101]. Despite the diverse host range and the fact that South Africa has the highest reported prevalence of *Trichinella* in sub-Saharan Africa [54], no human cases have been reported from South Africa to date.

*Trichinella* spp. infection is notifiable and listed in the Terrestrial Animal Health Code of the World Organization for Animal Health [58]. Owing not only to its potential economic and public health impact as a food-borne parasitic zoonosis, the diverse nature of the genus and subsequently diverse host range has led to a myriad of investigations aimed at elucidating not only its evolutionary expansion [80] but also the host-parasite relationships that exist within different ecological niches [64, 66, 70, 75, 78]. Factors influencing these relationships, however, are equally diverse and preclude any definitive report on the epidemiology of any one *Trichinella* species, especially where the natural sylvatic cycles are concerned.

The GKNP of South Africa represents a protected area where the abundance of sylvatic host species ensures both *Trichinella* spp. survival and transmission [43]. Scholtz et al. [88] reported that 1982 plant, 517 bird, 147 mammal and 21 reptile species exist in the approximate 20,000 km^2^ of the Kruger National Park (KNP) of South Africa. Several pieces of private land are additionally included by proclamation as part of the protected area, adding approximately another 37,430 ha which, collectively is known as the GKNP [88]. This species-rich and diverse habitat is maintained by intricate prey-predator-scavenger interactions, all of which are well protected within its borders. This creates an optimal system for species of the genus *Trichinella* to thrive.

However, the situation in the KNP is not unique and similarly optimal conditions may be expected in other national protected areas in sub-Saharan Africa such as the Serengeti (Tanzania), Kafue (Zambia), Hwange (Zimbabwe) and Gorongosa (Mozambique).

In this study, we reviewed published information on *Trichinella* infection in wildlife in the GKNP of South Africa from 1964 to 2019 and based on the results, the authors constructed complete hypothetical transmission cycles for the three taxa known to circulate in the GKNP. In justifying the hypotheses, the factors which may be influencing the establishment of these cycles are discussed, together with the potential of spillage into domestic environments and risk for human infections.

Pozio [65] noted differences in infection between host species as a result of unique host characteristics including diet, life span, distribution, behaviour and human interaction. Gottstein et al. [19] additionally noted that the survival of encysted larvae in host musculature is also influenced by host immunity, ultimately influencing the overall epidemiology of infection. Similarly, specific evolutionary adaptations among individual species of the genus affect their infectivity to specific hosts as well as epidemiology and survival in specific environments [64, 80]. These factors cannot be considered as constant either and are continually changing; most notably as a result of human activity and interaction, which influences environments, host species and parasites alike [12, 85]. However, these changes are, for the most part, slow in development, allowing at least some consistency as far as parasite transmission cycles are concerned. This allows for the elucidation of current epidemiology of *Trichinella* infections and more importantly, the prediction of probable host-parasite cycles within a set ecological niche.

These host-parasite interactions are likely to be more constant in environments such as national parks and wildlife protected areas where established relationships remain relatively unchanged through minimal human interference. This is especially true for *Trichinella* infections that evidently have a larger biomass in sylvatic animals compared to domestic animals [75].

## Materials and methods

### Study area

**Figure 1:**
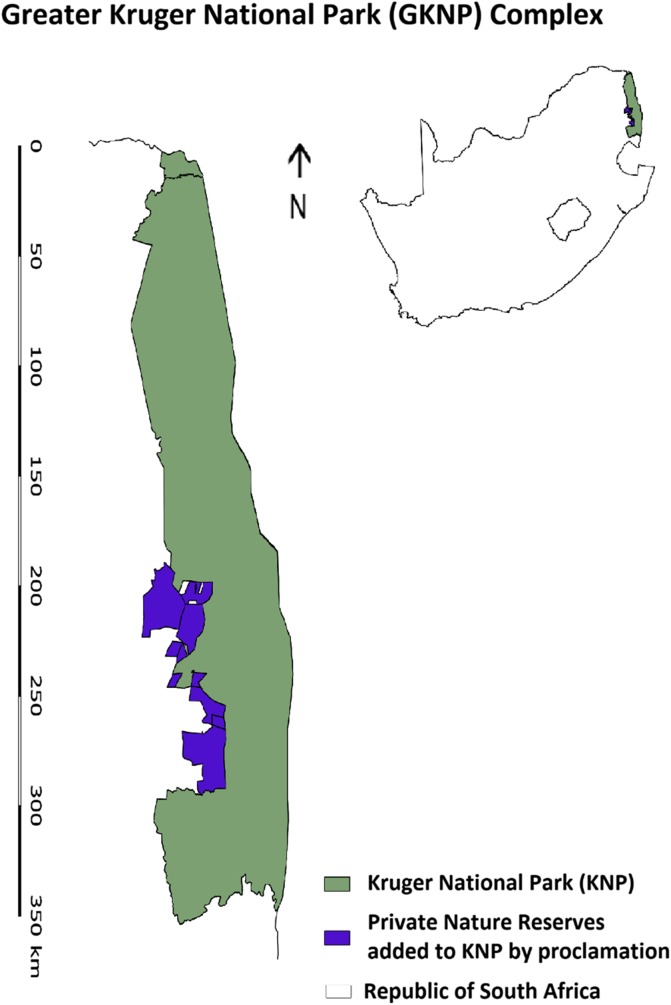
Map showing the Greater Kruger National Park of South Africa.

The KNP (Fig. 1) is situated in the North-Eastern corner of South Africa and is bordered by Zimbabwe to the north and Mozambique to the east [87]. This protected area covers almost 2 million hectares and boasts a diverse fauna comprising among others more than 150 mammal, 500 bird and 116 reptile species inhabiting its diverse tropical to sub-tropical “Biological Environment” [87]. The western- and south-western borders of the park are flanked by large communal areas and several private nature reserves while the southern border is mainly flanked by private agricultural and game farms. The impressive size of the GKNP allows for interactions between large predator and prey species which can be considered “near-natural” [87]. These conditions have undoubtedly favoured, specifically in respect of *Trichinella* spp. infections, the establishment and maintenance of unique parasite-host relationships.

### Search strategy

A search in Google Scholar, PubMed, AJOL and EBSCO Host database was conducted using the following terms and Boolean operators (AND, OR): *Trichinella* AND Wildlife; *Trichinella* infections in wildlife AND Kruger National Park; *Trichinella* spp, *Trichinella zimbabwensis*, *Trichinella* T8, *Trichinella nelsoni* AND Kruger National Park. Search results were carefully scrutinized and the relevant articles were selected for inclusion in the study. Some of the references of identified articles were additionally used to check for other relevant articles. The inclusion criteria included “all published peer-reviewed articles reporting on *Trichinella* infection in wildlife/livestock/humans in the GKNP from 1964 to 2019”.

#### Construction of hypothetical transmission cycles

The probability of *T. nelsoni*, *Trichinella* T8 and *T. zimbabwensis* parasites being transmitted among different wildlife hosts present in the GKNP was inferred from published literature on dietary habits of specific host species (Tables 1 and 2). No absolute or quantitative values could be attributed to these probabilities by any statistical means. The multifactorial and constantly changing nature of the multitude of ecological factors that may influence host interactions and subsequent parasite epidemiology precludes such an analysis. Furthermore, reports and publications from other sub-Saharan countries involving similar host species were also reviewed to provide supplementary data for the information portrayed in the hypothetical transmission cycles (Table 3).

**Table 1:** Predation/scavenging habits of wildlife species reported to harbour *Trichinella* spp. in sub-Saharan Africa (Events of predation/scavenging among species depicted below are not indicative of any degree of probability but merely suggest a possibility of such events occurring based on the literature cited).

Animal species	Common Name	Species predated/scavenged	References
*Panthera leo*	Lion	Warthog	[10, 21, 61]
		Rodents	[10]
		Baboon	[7, 21]
		Hyaena	[63, 84]
		Leopard, wild dog, cheetah	[63]
		Crocodile	[92]
		Lion	[57]
*Panthera pardus*	Leopard	Hyaena, lion, wild dog, cheetah	[63]
		Crocodiles	[63, 92]
		Baboon	[7, 27]
		Rodents	[26]
*Varanus niloticus*	Nile monitor	Rodents, juvenile crocodiles	[95]
*Crocuta crocuta*	Spotted hyaena	Warthog, baboon	[20]
		Lion	[63, 84]
		Leopard, cheetah	[63]
*Felis silvestris lybica*	African Wildcat	Rodents, carrion (unspecified)	[24]
*Canis mesomelas*	Black-backed jackal	Cheetah	[63]
		Rodents, carrion (unspecified)	[3, 5, 23]
*Civettictis civetta*	African civet	Rodents, carrion (unspecified)	[5]
*Genetta genetta*	Small spotted genet	Rodents	[39]
*Papio ursinus*	Chacma baboon	Baboon	[62]
		Rodents	[1]
*Praomys natalensis*	Multimammate mouse	Multimammate mouse	[26]
*Crocodylus niloticus*	Nile crocodile	Leopard	[63]
		Crocodile	[92]
		Nile monitor	[95]
		Lion, hyaena, warthog, baboon	[16]
*Potamochoerus larvatus* a	Bushpig	Carrion (unspecified)	[91]
*Phacochoerus africanus* a	Warthog	Hyaena	[84]
		Civet, carrion (unspecified)	[9]
*Canis adustis* a	Side-striped jackal	Rodents	[3, 5]
*Acinonyx jubatus* a	Cheetah	Rodents, carrion (unspecified)	[47]
*Leptailurus serval* a	Serval	Warthog	[82]
		Rodents, small spotted genet	
*Otocyon megalotis* a	Bat-eared fox	Rodents	[96]
*Ichneumia albicauda* a	White-tailed mongoose	Rodents	[11]
*Hyaena hyaena* a	Striped hyaena	Rodents, carrion (unspecified)	[98]

a Species native to GKNP and known host of *Trichinella* spp. elsewhere.

**Table 2:** Occurrence of *Trichinella* spp. in wildlife species from the Greater Kruger National Park, South Africa, from 1964 to 2019.

Animal species	Common name	No positive/tested	Total prevalence (%)	Tz	Tn	T8	NID	References
*Panthera leo*	Lion	22*/98	22.4	4	4	4	11	[36, 40, 43, 54, 79, 100]
*Panthera pardus*	Leopard	2*/7	28.6	1	1	1	0	[38, 54, 101]
*Varanus niloticus*	Nile monitor	1/2	NC	1	–	–	–	[54]
*Crocuta crocuta*	Spotted hyaena	17/26	65.4	2	–	1	14	[54, 100]
*Felis silvestris lybica*	African Wildcat	1/1	NC	–	–	–	1	[54]
*Canis mesomelas* **	Black-backed jackal	1/2	NC	–	–	–	1	[100]
*Civettictis civetta*	African civet	1/2	NC	–	–	–	1	[54, 101]
*Genetta genetta*	Small spotted genet	1/2	NC	1	–	–	0	[54]
*Papio ursinus*	Chacma baboon	1/6	16.7	–	–	–	1	[54]
*Praomys natalensis*	Multimammate mouse	1/44	2.3	–	–	–	1	[100]
*Crocodylus niloticus*	Nile crocodile	16/43	37.2	16	–	–	–	[35, 37, 54]
*Asio capensis*	Marsh owl	1/1	NC	–	–	–	1	[54]
Total		65/234		25	5	6	31	

Tz = *Trichinella zimbabwensis*, Tn = *Trichinella nelsoni*, T8 = *Trichinella* genotype T8, NID = Not identified to species level;

* One animal represents a mixed infection of *Trichinella nelsoni* and *Trichinella* T8;

** Incorrectly reported as Side striped jackal (*Canis adustus*) by Marucci et al. [43] and Mukaratirwa et al. [53], NC = Not calculated due to sample size < 5.

**Table 3:** Occurrence of *Trichinella* spp. in wildlife species from sub-Saharan Africa other than Kruger National Park, South Africa.

Country of origin	Animal species	Common name	No positive/tested	Total prevalence (%)	Tz	Tn	T8	Tb	NID	References
Tanzania	*Panthera leo*	Lion	3/24	12.5	–	3	–	–	–	[67]
Namibia			1/1	NC	–	–	1	–	–	[40], ITRC
Tanzania	*Panthera pardus*	Leopard	1/3	NC	–	1	–	–	–	[67]
Kenya			1/4	NC	–	1	–	–	–	[56, 66]
Zimbabwe	*Varanus niloticus*	Nile monitor	6/29	20.7	6	–	–	–	–	[53, 68]
Tanzania	*Crocuta crocuta*	Spotted hyaena	3/13	23	–	3	–	–	–	[67]
Congo			1/1	NC	–	–	–	–	1	[101]
Kenya			1/1	NC	–	1	–	–	–	[66], ITRC
Namibia			1/?	NC	–	–	–	–	1	[79]
Senegal	*Canis adustis*	Side-striped jackal	1/10	10	–	–	–	–	1	[18]
Kenya			?	NC	–	–	–	–	–	[66]
Namibia	*Canis mesomelas*	Black-backed jackal	1/?	NC	–	–	–	–	1	[79]
Tanzania			1/11	9	–	–	–	–	1	[86]
Senegal	*Ichneumia albicauda*	White-tailed mongoose	6/17	35.3	–	–	–	–	6	[18]
Guinea	*Civettictis civetta*	African civet	1/19	5.3	–	–	–	1	–	[78]
Guinea	*Nandinia binotata*	African palm civet	2/45	4.4	–	–	–	1	–	[78]
Kenya	*Potamochoerus larvatus*	Bush pig	1/40	2.5	–	–	–	–	1	[56]
Kenya	*Phacochoerus africanus*	Warthog	18/450	4	–	–	–	–	18	[18]
Tanzania			1/1	NC	–	1	–	–	–	[38], ITRC
Zimbabwe	*Crocodylus niloticus*	Nile crocodile	256/648	39.5	256	–	–	–	–	[15]
Mozambique			8/40	20	8	–	–	–	–	[68]
Tanzania	*Acinonyx jubatus*	Cheetah	1/5	20	–	1	–	–	–	[67]
Kenya	*Leptailurus serval*	Serval	1/9	11	–	1	–	–	–	[56, 66]
Tanzania	*Otocyon megalotis*	Bat-eared fox	1/6	17	–	1	–	–	–	[67]
Kenya	*Hyaena hyaena*	Striped hyaena	1/2	50	–	1	–	–	–	[56, 66]
Nigeria	*Cricetomys gambianus*	African giant rat	16/100	16	–	–	–	–	16	[46]
Nigeria	*Sus domesticus*	Domestic pigs	42/883	4.8	–	–	–	–	42	[2]
	Total		379/2362	16	270	15	1	2	88	

Tz = *Trichinella zimbabwensis*, Tn = *Trichinella nelsoni*, T8 = *Trichinella* genotype T8, Tb = *Trichinella britovi*, NID = Not identified to species level, NC = Not calculated/reported due to sample size < 5. ITRC = International *Trichinella* Reference Centre, ? = Actual numbers not reported in cited literature.

Based on the available prevalence data, lions are proposed to be the main reservoirs for both *T. nelsoni* and *Trichinella* T8, while crocodiles are considered to be main reservoirs for *T. zimbabwensis*. However, there is limited information on additional and other potential reservoirs, and in most cases the numbers of animals screened for *Trichinella* spp. infection are very low. It is also important to consider the overall biomass of each potential host species within the ecological framework being assessed. Species representing a larger biomass will require a higher number of individuals to be tested compared to species with a smaller biomass in order to reach conclusive evidence in respect of identifying main reservoirs. In the case of rodents, this problem is further compounded by the fact that vertical parasite transmission is possible via both the transmammary and transplacental routes [45].

## Results

### *Trichinella* species reported in the GKNP

From the beginning of *Trichinella* surveillance studies in South Africa in 1964 to the end of 2016, at least two species and one genotype have been confirmed across six mammalian and two reptilian hosts from the GKNP [38, 40, 43, 54, 79]. *Trichinella*-like infections have additionally been reported in six other mammalian hosts, but species confirmation of the parasite was not possible [43, 54, 100, 101]. The unidentified isolates, for the most part, were generally believed to be one or more of the parasite species known to circulate in the area. A *Trichinella*-like infection has also been reported in a Marsh owl (*Asio capensis*), possibly suggesting the existence of an additional *Trichinella* specie not known to occur on the African continent or a different tissue-dwelling nematode/larva not related to *Trichinella* [54] (Table 2).

In the GKNP, the prevalence of *T. nelsoni* in lions and hyaenas was reported to be 3/98 (3.06%) and 0/26 (0%), respectively [54]. It is important to note that the majority of these isolates (11/21) and (14/17) found in lions and hyaenas respectively, were not identified to species level [54] and thus the actual prevalence in GKNP could be higher than reported. Based on the overall prevalence of *T. nelsoni* in sub-Saharan Africa (Table 3) and the GKNP (Table 2), hyaenas and lions are considered to be the main reservoirs for this parasite species in the GKNP, as may be the general case with similar habitats in other African countries [43, 53].

In the GKNP, only a single leopard (1/7, 14%) tested positive for *T. nelsoni* [54] and the same species has previously been isolated from leopards in Kenya [66] and Tanzania [67]. La Grange et al. [38] described a mixed infection of *T. nelsoni* and *Trichinella* T8 in a leopard from the GKNP and based on the dietary habits of the species [4, 20, 42, 94], we hypothesise that other small mammalian carnivores may serve as an important source of infection to these animals in the GKNP.

Similar to *T. nelsoni*, genotype *Trichinella* T8 has been found in low prevalence in lions (4/98, 4%), hyaenas (1/26, 3.8%) [43, 54, 76] and leopards (1/7, 14%) from the GKNP [38, 54]. Again, as in the case of *T. nelsoni*, many *Trichinella* spp. isolates found were reported prior to the advent of molecular characterisation techniques, and thus the parasite species involved remain unknown. Data on the actual distribution and prevalence of *Trichinella* T8 are still fragmented. Although closely related to *Trichinella* T8, *Trichinella britovi* has never been isolated from South African wildlife. Pozio and Murrell [75] confirmed the geographical distribution of *T. britovi* to include amongst others Northern and Western Africa, whereas *Trichinella* T8 is confined to the South Western and South Eastern parts of Africa. Pozio et al. [78] hypothesized that large natural barriers such as the Zaire lake basin and river Cross of Nigeria, together with environmental changes, may have contributed to the evolution of these two unique taxa.

*Trichinella zimbabwensis* was previously isolated from wild Nile crocodiles (*Crocodylus niloticus*) in the KNP and just beyond its north-western- and southern boundaries [35, 37, 54], and in a Nile monitor lizard (*Varanus niloticus*) from the city of Nelspruit located close to the south-western border of the KNP [54]. Furthermore, it has also been detected in farmed crocodiles in South Africa (Department of Agriculture, Forestry and Fisheries (DAFF), personal communication). This species is infective to mammals and reptiles [51–53, 74]. Results from passive surveillance in the GKNP further revealed that *T. zimbabwensis* has the highest prevalence in crocodiles and carnivores, of three species known to circulate in South Africa [54].

### Hypothetical transmission cycles of *Trichinella* spp. in GKNP

Previous findings have prompted speculation concerning the epidemiology of *T. nelsoni*, *Trichinella* T8 and *T. zimbabwensis*, including hypothetical transmission cycles as proposed by Mukaratirwa et al. [53]. Since the publication of these hypotheses, new host species have been confirmed [54], prompting a revision of the proposed hypotheses. Unravelling the enigmatic epidemiology of these potentially zoonotic species from the genus *Trichinella* is important from a public health perspective as it aids in establishing not only the potential risk for human infection [63], but ultimately proper control and prevention measures [53, 64, 80].

**Figure 2:**
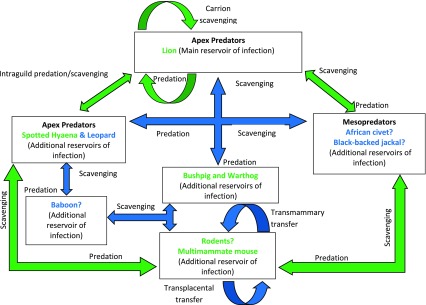
Updated hypothetical sylvatic cycle of *Trichinella nelsoni* and *Trichinella* T8 in the Greater Kruger National Park (GKNP) of South Africa. ? = Species involvement is yet to be confirmed; Arrows indicate direction of transmission; Arrows in green colour = Previously hypothesised mode of transmission (Mukaratirwa et al. [53]); Arrows in blue colour = Additional hypothesised mode of transmission (current hypothesis); Host species in green text = Previously hypothesized host species (Mukaratirwa et al. [53]); Host species in blue text without ? = Additional host(s) species (current hypothesis).

**Figure 3:**
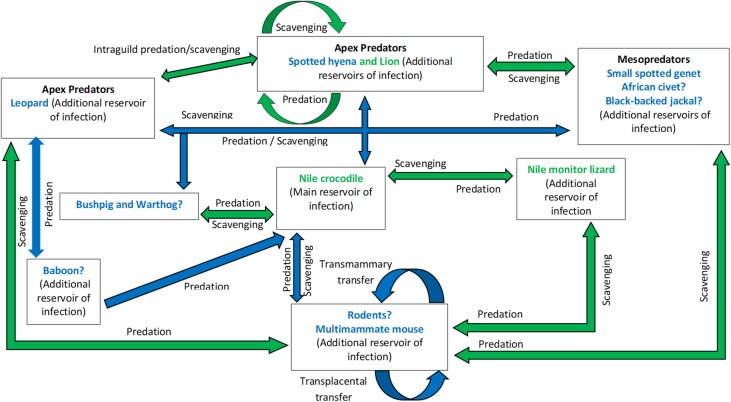
Updated hypothetical sylvatic cycle of *Trichinella zimbabwensis* in the Greater Kruger National Park (GKNP) of South Africa. ? = Species hypothesized to be involved but yet to be confirmed; Arrows indicate direction of transmission; Arrows in green colour = Previously hypothesised mode of transmission (Mukaratirwa et al. [53]); Arrows in blue colour = Updated hypothesised mode of transmission (current hypothesis); Host species in green text = Confirmed host species (Mukaratirwa et al. [53]); Host species in blue text without ? = Updated confirmed host(s) species (current hypothesis).

New additions to the knowledge on the prevalence of *Trichinella* spp. isolated from wildlife hosts in the GKNP and other surrounding areas outside the park and elsewhere in Eastern and Southern Africa provides for an update of the previously hypothesised transmission cycles for the three taxa known to circulate in this area. Considering the potential epidemiological drivers based on host species richness and interspecies interaction in the GKNP and the region discussed earlier, hypothetical transmission cycles for the three *Trichinella* taxa are proposed in Figures 2 and 3.

Based on the sympatric existence of *T. nelsoni* and *Trichinella* T8, we hypothesize a transmission cycle applicable to both these species (Fig. 2). The hypothetical cycle previously presented by Mukaratirwa et al. [53] was updated to include recent findings presented by Mukaratirwa et al. [54]. A separate hypothetical cycle is presented for *T. zimbabwensis* (Fig. 3), and was similarly updated from Mukaratirwa et al. [53] to include recent findings [54]. Two apex predators (hyaena and leopard) and a mesopredator, the small spotted genet (*Genetta genetta*) have been confirmed as new host species and included in the hypothetical transmission cycle. Additionally, rodents and in particular the multimammate mouse (*Praomys natalensis*) [100] and three mesopredators, the African civet (*Civettictis civetta*) [101], black-backed jackal (*Canis mesomelas*) [100] and African wild cat (*Felis silvestris lybica*) [54], which were previously found to be infected by unidentified species of *Trichinella*, have been added as probable host species in both hypothetical cycles.

Interspecies predation between hyaenas and lions has previously been presented as a contributing factor in the maintenance of the two encapsulated *Trichinella* species, *T. nelsoni* and *Trichinella* T8 found in GKNP [53]. Results reported by Mukaratirwa et al. [54] now also suggest that *T. zimbabwensis* could be similarly maintained between these species. The addition of a leopard as a host for *T. zimbabwensis*, however, compels its inclusion into this equation. However, several factors that drive intraguild predation as discussed by Palomares and Caro [60] need to be taken into account. The interactive role of leopards may be predominantly asymmetrical with leopards being more prone to predation by the other two species compared to a more symmetrical interaction between lions and hyaenas. However, as predators, leopards play a much more significant role in *Trichinella* epidemiology when considering their interaction with smaller mesopredators, such as the small spotted genet. Mukaratirwa et al. [54] also alluded to the importance of mesopredators as sources of infection to larger species and the possible existence of a large parasite biomass in rodents and reptiles that could act as a primary infection source.

## Discussion

Parasites of the genus *Trichinella* are known to primarily infect sylvatic carnivores with cannibalistic and/or scavenger behaviour [9, 53, 75]. Domestic cycles involving some species, most notably *T. spiralis* are recognised [19, 64] and intrusion from the sylvatic cycle into the domestic environment usually results from human failure to properly manage the wildlife-domestic animal interface [75]. Pozio [64] noted that successful intrusion from the sylvatic cycle and the subsequent maintenance of the flow of parasites between sylvatic, synanthropic and domestic environments relies on parasite and ecological characteristics, human behaviour and availability of synanthropes. This would ultimately result in unique life cycles for each taxon within a specific ecological niche. *Trichinella nelsoni* is known to occur in Eastern and Southern Africa [53, 78] and has been detected in Kenya, Tanzania and South Africa [38, 43, 54, 67, 79]. Carnivores appear to be the major reservoirs for *T. nelsoni* and the parasite has been found in high prevalence especially in lions (*Panthera leo*) (35.3%) and spotted hyaenas (*Crocuta crocuta*) (29.4%) [53]. It has additionally been detected in leopards (*Panthera pardus*) [38, 67], cheetahs (*Acinonyx jubatus*) and bat eared foxes (*Otocyon megalotis*) [67].

Despite high infectivity to carnivores, members of the Suidae family are only moderately susceptible to *T. nelsoni* [28, 29]. These findings are supported by the fact that the actual prevalence of *Trichinella* spp. in wild Suidae appears to be very low [53, 75]. Despite the reported low infectivity of wild Suidae to *T. nelsoni* [28, 29], previous reports of infections in these animals [18, 56, 75, 79, 86] suggest that they might, albeit to a lesser extent, play a role in the epidemiology of *Trichinella* spp. in the GKNP. Importantly, the aforementioned was discovered prior to the advent of molecular characterisation techniques, and thus the parasite species involved remain unknown although the involvement of *T. nelsoni* cannot be ruled out. The exception might be the studies by Grétillat and Chevalier [18], which were conducted in Senegal where the reported infections may have involved *T. britovi*.

In the Majete Wildlife Reserve in Kenya, interspecies cannibalism among warthogs (*Phacochoerus africanus*) was observed and predation by warthogs on hyaena cubs suggested as a contributing factor to the small hyaena population [84]. Apart from the incidences reported by Sachs [86], *Trichinella* spp. infection has never been reported in wild pigs from South Africa albeit that the numbers screened thus far have been very low [54].

*Trichinella* T8 has previously been isolated in a lion from the Etosha National Park in Namibia [75, 79], but surprisingly has never been positively identified in any other African country except South Africa [53, 66]. However, Marucci et al. [43] did observe that both *T. nelsoni* and *Trichinella* T8 appear to circulate among hyaenas and lions in the KNP with similar prevalence and hypothesized sympatric status. This sympatry is confirmed to extend to other host species and include leopards [38].

Results from passive surveillance in the GKNP revealed that *T. zimbabwensis* has the highest prevalence in Nile crocodiles and carnivores compared to the three species known to circulate in South Africa [54]. *Trichinella zimbabwensis* has also been detected in farmed crocodiles in South Africa (DAFF, personal communication). This parasite species was proved experimentally to be infective to mammals and reptiles [51–53, 70] and notably, domestic pigs [44, 52]. No known cases of human disease have been associated with *T. zimbabwensis*. However, its infectivity to pigs and other animal species utilized as human food sources provide the incentive to regard this species as a high risk species of food safety concern [13].

Moleón et al. [48] suggested the risk of parasitic infection associated with conspecific and heterospecific carrion scavenging between carnivores to be a selective force preventing carnivores from eating each other. However, in the case of *Trichinella* infections, predation and scavenging among carnivores is the primary mode of transmission and carnivores are the primary hosts [8]. Carnivores like lions and hyaenas are considered apex predators [60] and have a dietary overlap of more than 68% [20, 61]. Additionally, within the GKNP, these two predators both prefer the same habitat [61], which may result in encounters where kleptoparasitism by both species frequently occurs [41]. These encounters can prove fatal to individuals of both species although mortality of hyaenas is usually higher [61]. There is a paucity of literature on the actual incidence of intraguild predation with consumption of the victim by these two predators. Palomares and Caro [60] have shown that carnivores sometimes consume or at least partially consume their victims. This would suggest intraguild scavenging between carnivores to be secondary to active predation where the transmission of *Trichinella* spp. is concerned. However, secondary carrion scavenging by both apex- and mesopredators such as jackals (*Canis mesomelas*) on carnivore carcasses cannot be totally excluded.

Leopards (*P. pardus*) are known to prey on smaller mesopredators [4, 22, 42] and a review by Palomares and Caro [60] showed that these opportunistic predators not only killed but consumed a variety of carnivore species, including young hyaenas [4]. Similarly, lions and hyaenas frequently kill and sometimes consume smaller mesopredators [60].

A previous study showed that odours, specifically 2-phenylethylamine, from carnivore carcasses trigger an innate fear response that leads to avoidance of carnivore carrion by rodents [14]. However, multimammate mice (*P. natalensis*) are known to occur in the GKNP and their diet can include carrion [49]. This is also supported by the fact that a single case of *Trichinella* infection was previously reported in this species from the KNP [100]. Vertical transmission of *T. zimbabwensis* via the transmammary and transplacental routes has been experimentally proven in rodents (*Rattus norvegicus*) [45], which suggests that endemic rodent populations in the GKNP may play an integral role in the maintenance and transmission of the three *Trichinella* taxa known to circulate in the region.

In addition to the plethora of potential mammalian hosts, the GKNP is home to a high population of Nile crocodiles (*C. niloticus*) and predation between crocodiles and mammalian carnivores is known to occur [99]. Previous studies have shown a high prevalence of *T. zimbabwensis* in Nile crocodiles in the KNP [35]. This could probably be attributed to high levels of intraspecies predation and scavenging among crocodiles. However, a recent report by Mukaratirwa et al. [54] showed *T. zimbabwensis* not only to be the most prevalent, but also to infect the widest host range of all the *Trichinella* species isolated thus far from the GKNP. This would certainly suggest the general knowledge and perceptions of interspecies predation and scavenging to be incomplete.

### Limitations of the review

Several factors preclude a co-ordinated surveillance effort to enable screening of all the potential host species in the GKNP and other nature reserves in the rest of South Africa and elsewhere. Access to a variety of samples is reliant on the acquisition of convenient samples from State Veterinary Services, reserve staff and private veterinarians. A more structured and co-ordinated approach such as the effective implementation of existing regulations [Regulation (EU) 2015/1375] employed in North America, Europe and Asia is required to maintain and improve wildlife surveillance for *Trichinella* infections in GKNP.

All potential stakeholders should be sensitized to the importance of surveillance through continued collaborative efforts. Many of the potential host species are also protected by national and international legislation, which further hampers sample acquisition. Overcoming legislative barriers can only be attained through close collaboration with local authorities. Establishing effective communication between researchers and other stakeholders with the applicable authorities mandated to regulate the collection and transportation of samples is essential to future success. Lack of funding and other resources also precludes effective surveillance. Private and institutional funding opportunities should continually be sought and motivated through highlighting the potential impact of *Trichinella* on human health and the threat to commercial farming industries.

Indeed, the lack of data on human infections and cases involving domestic animals has resulted in *Trichinella* surveillance not being considered a public health priority by the controlling veterinary authority. This perception needs to be changed and emphasis must be placed on the marginal cost of surveillance compared to the cost of remedial action in the event of a human outbreak, or the cost of control and eradication in the event of domestic spill-over.

### Research gaps and future research

Maintenance of, and where possible, improvement of collaborative efforts with GKNP staff and other stakeholders is crucial. Wildlife surveillance should also be encouraged in other African countries and the rest of South Africa, and hence there is need to employ current knowledge and expertise to establish a *Trichinella* Reference Centre for Africa to assist in the surveillance of infections and capacity building of expertise.

A study on the role of predatory fish as potential hosts for *T. zimbabwensis* is currently underway in South Africa. A previous study suggests that fish do not play any significant role in the epidemiology of *T. zimbabwensis* [72]. However, the potential host species used in the study are not associated with either Nile crocodiles or Nile monitor lizards in nature and do not co-exist with any of the predators in any of their respective natural habitats. Previous studies have shown that host characteristics play an important role in determining not only muscle predilection but also the infectivity of different *Trichinella* species to different hosts [30, 31, 37, 83, 93]. Studies with fish experimentally infected with encapsulated *T. britovi* and *T. spiralis* and non-encapsulated *T. pseudospiralis* did show that the larvae, even though they did not develop into adults, migrated to the body cavity and internal organs (*T. spiralis*) and also the muscles (*T. britovi* and *T. pseudospiralis*) of some fish species and retained their infectivity for a limited period of time [50, 97]. However, interspecies differences between parasites of the genus *Trichinella* have also been shown to influence both muscle predilection and infectivity in the same host [25, 32, 33]. In order to fully understand parasite epidemiology, the correct selection of a probable host species and parasite species based on their natural occurrence is of utmost importance.

Future surveillance efforts will also include more focused efforts on migratory carnivorous birds and targeted surveillance of rodents to elucidate their potential role as maintenance reservoirs for the different *Trichinella* taxa in GKNP.

## Conclusion

The vast size and limited human interference combined with the species richness within the protected area of the GKNP provide an excellent setting for the establishment and maintenance of the *Trichinella* spp. known to circulate in the area. As a testament to this, *T. zimbabwensis*, *T. nelsoni* and *Trichinella* T8 have all established very unique and diverse transmission and maintenance cycles consisting of a multitude of equally diverse host species. Results from surveys spanning more than 50 years suggest that our knowledge of the actual incidence and epidemiology of *Trichinella* in this area is curtailed at best. As such, the information presented here cannot, by any means, be considered complete but should rather be viewed as ongoing which undoubtedly will require future update as new evidence is presented. Despite much of the information presented being based on anecdotal evidence, this study confirms not only a need for more intense epidemiological surveillance in the rest of South Africa and beyond [53], but also the need for continued efforts to unravel the remaining gaps in the epidemiology of *Trichinella* spp. in these unique and diverse protected landscapes in eastern and southern Africa.
